# Diet

**Published:** 1906-04-21

**Authors:** 


### Hospital Clinics.

### DIET.

Dr. Harry Campbell [1] prefaces a recent lecture upon this subject with the question: "How far is man to be regarded as a vegetable feeder, and how far as an animal feeder?" The mammalian branch of the vertebrate animal kingdom falls, in respect of diet, into three classes: (1) the purely flesh feeders, the carnivora; (2) the purely vegetable feeders, which include the herbivora and many of the frugivora; and (3) the mixed feeders. The herbivora, such as the ox, horse, and rabbit, subsist upon a bulky unconcentrated food—grasses, leaves, etc.—and have a correspondingly bulky digestive system. The frugivora, on the other hand, live on a far more concentrated diet and have in consequence a digestive system far less bulky in proportion to their size than the herbivora. Last come the mixed feeders, such, for instance, as squirrels, which supplement their vegetable diet with eggs, and monkeys, many of which devour small birds and lizards and grubs.

To which of these three great classes does man belong? The author thinks that there can be no doubt as to the answer. Man is naturally a mixed feeder, like the existing anthropoid apes, the gorilla, orang outan, gibbon, and chimpanzee. "We have, beyond all doubt, descended from a being closely allied to these creatures, and we are therefore justified in assuming that the diet of our simian ancestors was much the same as that of the present-day anthropoids, a conclusion rendered all the more certain by the remarkably close resemblance between their digestive organs and our own." Dr. Campbell considers it certain, from observations upon the anthropoid apes in captivity, and from the records of travellers, that the anthropoids are essentially frugivorous, but at the same time depend to a certain extent upon animal food, such as birds, snakes, lizards, eggs, insects, and grubs.

Passing from this consideration of the anthropoids, the author deals with man in the "pre-cibicultural" phase of culture, that is to say, a phase in which no effort is yet made to cultivate the vegetable world for the purposes of food, or to breed animals with a similar object. The pre-cibiculturists live, therefore, upon wild fruits, seeds, roots, and such animal food as they can procure by hunting and fishing. Such peoples still exist. The Esquimaux, for instance, and certain acorn-eating Californian tribes, the former of whom are, from force of circumstance, almost entirely flesh-feeders, while the diet of the latter is about two-thirds vegetable and one-third animal. Thus, in spite of a knowledge of cookery which enables them to extend widely the category of vegetable substances which can be included in their dietary, the pre-cibiculturists remain essentially mixed feeders; it is fairly certain, therefore, that man in the days before the evolution of cooking was not less dependent upon the animal world than the most primitive existing types of the present day.

The conclusion, therefore, is that man must have steadily increased in carnivorism from his anthropoid stage until the time when he began to prepare his vegetable food in special ways. He was a hunter and a fisher, and not until the period of fixed agriculture, when he became rooted to a more or less defined area of the soil, did these two occupations cease to be the serious business of his life. He was thus most carnivorous at an epoch when his life was of the most active and out-of-door character. Even the naturally pure carnivores, such as the dog, when kept artificially to a sedentary and confined existence cannot tolerate without illness more than a small quantity of concentrated animal food.

Reducing this thesis to terms of practice, the author affirms that if the question be asked: "What food is best suited to man's requirements—animal, vegetable, or mixed?" an unhesitating answer can be given to the effect that a mixed diet is so best suited, the proportions of animal to vegetable varying with the mode of life of the individual concerned. Now, although we are forced to conclude that man is by inheritance largely carnivorous, there is no doubt that the capacity to tolerate flesh foods manifests a great variability in different individuals. A few people thrive best upon a diet that is entirely, or almost entirely, vegetable; these are to be looked at as exceptions, as reversions to a far-off ancestral type. There is no doubt, in the author's opinion, that good can sometimes be effected, especially in the case of the gouty, by curtailing animal food or entirely excluding it from the dietary. None the less, a purely vegetarian diet is for man an unnatural one.

How much, in the next place, ought a man to eat of these mixed foods? The ideal quantity, as stated by the author, is what he terms "the minimum normal," that is to say, "the smallest quantity of food adequate to maintain a man at the lowest weight compatible with the highest attainable level of health." Any quantity above this is useless, if not actually harmful. In this matter there are wide personal differences, for many people well on in years can tolerate with seeming impunity a quantity of food far in excess of the minimum normal. Dr. Campbell thinks, however, that these individuals pay for their gluttony by a degree of health which falls short of the highest obtainable. But there is no question that occasionally gluttons live to a green old age.

Of the evils associated with over-eating, the most obtrusive is obesity, the existence of which postulates an immense amount of wasted labour thrown upon the heart; this consideration points the importance of reducing the weight of elderly and stout people, especially in the presence of evidence of cardiac weakness. The same argument applies to the obesity so often associated with bronchitis. Another evil effect is indigestion, which includes the burdening of the liver with an excess of imperfectly digested material, and a consequent bathing of the tissues in a plasma which is faulty both from "surcharge of nutriment and perversion of composition." The importance of food-excess depends upon the fact that such excess is not simply passed through the system unaltered—if it were, the dangers of over-eating would be reduced to mechanical ones—but all the excess of nutriment which is absorbed is metabolised in the tissues and discharged in the shape of urea, carbonic acid, water, and the like. This implies that over-eating means an increased demand for oxygen; it therefore means extra work not only for the digestive organs and kidneys, but also for the lungs and heart. These considerations supply indications for treatment in the case of acute dyspnœa, whether of cardiac or of pulmonary origin. A meagre diet is of the first importance in these conditions. Similarly, in the acute phase of specific fevers starvation is of great value on account of the relief afforded thereby to the circulatory system. When the heart is failing, much more good results from the reduction of its work by a process of starvation than from a conjunction of generous diet with liberal doses of alcohol intended to fit the heart for the extra work thus prepared for it.

In conclusion, the author insists that there is no fear in practice of starving a patient to death by such curtailment of food. "People do not so easily die of starvation as is generally thought. I have never myself seen anyone die from lack of food except when the digestive tract has been obstructed —*e.g.*, from malignant disease—or in the all too familiar instances of neglected children amongst the poor, and in these cases death generally results rather from giving the wrong kind of food than too little of it. On the other hand, I am certain that many lives may be saved by a judicious reduction of the diet, even to the point of temporarily withholding it altogether."

[1] Clin. Jour., March 14, 1906.

